# TMT-based quantitative proteomic analysis of spheroid cells of endometrial cancer possessing cancer stem cell properties

**DOI:** 10.1186/s13287-023-03348-x

**Published:** 2023-05-04

**Authors:** Mingzhu Cao, Zhi Liu, Danming You, Yingying Pan, Qingyan Zhang

**Affiliations:** 1 Department of Obstetrics and Gynecology, Center for Reproductive Medicine, Key Laboratory for Major Obstetric Diseases of Guangdong Province, The Third Affiliated Hospital of Guangzhou Medical University, No.63, Duobao Road, Guangzhou, China; 2 Key Laboratory for Reproductive Medicine of Guangdong Province, The Third Affiliated Hospital of Guangzhou Medical University, Guangzhou, China; 3 Department of Ultrasound, Nanfang Hospital, Southern Medical University, No.1838, Baiyun Road North, Guangzhou, China; 4 The First School of Clinical Medicine, Southern Medical University, Guangzhou, China; 5 Reproductive Medicine Center, The First Affiliated Hospital, Sun Yat-Sen University, No. 1, Zhongshan 2nd Road, Guangzhou, China; 6 Guangdong Provincial Key Laboratory of Reproductive Medicine, the First Affiliated Hospital, Sun Yat-Sen University, Guangzhou, China; 7 Department of Endocrinology and Metabolism, Nanfang Hospital, Southern Medical University, Guangzhou, China

**Keywords:** Cancer stem cell, Endometrial cancer, HIF-1 pathway, Quantitative proteomic, Spheroid cell

## Abstract

**Background:**

Cancer stem cells (CSCs) play an important role in endometrial cancer progression and it is potential to isolate CSCs from spheroid cells. Further understanding of spheroid cells at protein level would help find novel CSC markers.

**Methods:**

Spheroid cells from endometrial cancer cell lines, Ishikawa and HEC1A, exhibited increased colony forming, subsphere forming, chemo-drug resistance, migration, invasion ability and tumorigenicity, verifying their cancer stem-like cell properties. The up-regulated CD90, CD117, CD133 and W5C5 expression also indicated stemness of spheroid cells. TMT-based quantitative proteomic analysis was performed to explore the potential alterations between parent cells and cancer stem-like spheroid cells. HK2-siRNA was transfected to Ishikawa and HEC1A cells to explore the roles and molecular mechanism of HK2 in endometrial cancer.

**Results:**

We identified and quantified a total of 5735 proteins and 167 overlapped differentially expressed proteins of two cell types, 43 proteins were up-regulated and 124 were down-regulated in spheroid cells comparing with parent cells. KEGG pathway revealed a significant role of HIF-1 pathway in spheroid cells. qRT-PCR and western blot results of GPRC5A, PFKFB3 and HK2 of HIF-1 pathway confirmed their elevated expressions in spheroid cells which were consistent with proteomic results. HK2 promoted cancer stemness in endometrial cancer.

**Conclusion:**

These findings indicate that spheroid cells from endometrial cancer cell lines possess cancer stem-like cell properties and enrich CSCs. HIF-1 pathway is activated in endometrial cancer stem-like spheroid cells.

**Supplementary Information:**

The online version contains supplementary material available at 10.1186/s13287-023-03348-x.

## Introduction

Endometrial cancer (EC), accounting for estimated 12,940 women’s deaths and 66,570 new cases annually in the US, is becoming the most common gynecological tumor in the developed countries [1, 2]. The incidence has increased and death rates rose over the past decade. Although the exact mechanism remains unidentified, an emerging theory focusing on endometrial cancer stem cells (ECSCs) is rather notable [3–5].

Cancer stem cells (CSCs) are a group of tumor cells which share some characteristics with normal stem cells, including self-renewal, multi-potential differentiation and unlimited proliferation. CSCs were first found in acute myeloid leukemia, and then in some solid tumors [6, 7]. They are associated with chemotherapy resistance, endocrine therapy resistance, recurrence, invasion and metastasis of cancers [8–10]. The latest research also suggested that CSCs resided in endometrial carcinoma tissue, with the ability of colony forming and tumorigenicity [11].

Current ways to isolate CSCs mainly include fluorescence activated cells sorting (FACS) and magnetic activated cells sorting (MACS) based on surface markers expression, and side population (SP) cell sorting methods [12]. However, the specific markers of stem cells for endometrial cancer have not been verified, which makes the common separation methods difficult to conduct. Thus, reliable marker(s) of ECSCs are urgent to be identified in order to understand the cancer stem cells features.

Suspension cultivation is another method widely used to isolate CSCs. Cancer stem cells tend to proliferate and form into spheres that carrying the CSCs markers [13]. Precisely, spherical colonies have been considered to provide biological function and molecular characteristics of CSC in various solid tumors such as breast cancer, gastric cancer, prostate cancer, colorectal cancer and gliomas [14–16]. However, only a few studies reported the stem-like features of spheroids in endometrial cancer cells [17, 18]. These researches showed that some CSC-related markers such as CD133, CD44, ALDH1A1 and NANOG were upregulated and tumorigenicity was enhanced in spheroid cells of endometrial cancer. However, the comprehensive or detailed features of endometrial cancer spheroid cells have not been characterized.

Therefore, we hypothesized that spheroid cells from endometrial cancer carry certain markers and possess cancer stem-like cells features. In this study, we identified the stem-related markers and illustrated the features for further understanding of endometrial cancer spheroid cells. Subsequent proteomics analysis may also contribute to the identification of novel CSC markers and therapeutic approaches. TMT-based quantitative proteomic was first used to explore the potential markers or molecular mechanisms of CSCs in endometrial cancer spheroid cells and HIF-1 pathway was identified to be activated in spheroid cells enriching CSCs.

## Materials and methods

### Cell culture

The human endometrial cancer cell line Ishikawa was purchased from Procell Life Science Co. Ltd. (Wuhan, Hubei, China). HEC1A was preserved by our lab. Cells were grown in Dulbecco’s modified Eagle’s medium (DMEM; Gibco; Thermo Fisher Scientific, USA) containing 10% fetal bovine serum (FBS; Gibco, Thermo Fisher Scientific, USA) with 100 U/ml penicillin/streptomycin. The cells were maintained at 37 °C in a 5% CO_2_ incubator.

### Spheroid formation assay

The Ishikawa and HEC1A cells were seeded at a density of 3000 cells in 6-well ultralow-adherent dishes (Corning, USA) containing complete medium, and then cultured at 37 °C under 5% CO_2_ for 5 days. Formation of spheroids were observed and counted using an inverted microscope (Nikon, Japan). Spheroid cells were selected, isolated individually with pipette and real-time observation under the microscope, and they were grouped as spheroid cells. In contrast, the original cell lines were grouped as parent cells. The parent cells and spheroid cells were treated with trypsin to become single cell and seeded at a density of 3000 cells in 6-well ultralow-adherent dishes containing serum-free medium for subsphere-forming assay.

### RNA isolation and quantitative RT-PCR (qRT-PCR)

Total RNAs were extracted separately from the spheroid cells and parent cells using RNAiso Plus (Takara Biomedical Technology, Beijing, China) [19]. Then RNAs were reversely transcribed into cDNA using PrimeScript® RT reagent Kit (Takara Biomedical Technology, Beijing, China). The qRT-PCR assays were performed in Quant Studio 5 (Applied Biosystems, USA) using SYBR® Premix Ex TaqTM II Kit (Takara Biomedical Technology, Beijing, China). The sequences of qPCR primers of G protein coupled receptor, family C, group 5, member A (GPRC5A), 6-phosphofructo-2-kinase/fructose-2,6-bisphosphatase 3 (PFKFB3), and Hexokinase 2 (HK2) were detailed in Table 1. The 2^−ΔΔCt^ method was used to calculate the relative levels of the average of three replications.

**Table 1:** Details of PCR primers

Genes	Forward primer	Reverse primer
GAPDH	AGCCTTCTCCATGGTGGTGAAGAC	CGGAGTCAACGGATTTGGTCG
GPRC5A	CCAGGCATTCGGCAATGTG	CCACCGTTTCTAGGACGATGC
PFKFB3	TTGGCGTCCCCACAAAAGT	AGTTGTAGGAGCTGTACTGCTT
HK2	GAGCCACCACTCACCCTACT	CCAGGCATTCGGCAATGTG

### Flow cytometry

Stem cell markers of the spheroid cells and parent cells were detected by flow cytometry (FACScan, BD Biosciences, USA) [20].The spheroid and parent cells of Ishikawa and HEC1A were treated with trypsin and washed by phosphate buffer saline (PBS) twice. CD29 (anti CD29-PE, TS2/16, eBioscience, USA), CD44 (anti CD44-APC, IM7, eBioscience, USA), CD90 (anti CD90-FITC, eBio5E10, eBioscience, USA), CD105 (anti CD105-APC, SN6, eBioscience, USA), CD117 (anti CD117-PE, 104D2, eBioscience, USA), CD133 (anti CD133-PE, TMP4, eBioscience, USA), W5C5 (anti SUSD2-PE, W5C5, eBioscience, USA), and CD326 (anti CD326-FITC, TS2/16, eBioscience, USA) antibodies were added into the cell suspension respectively. After incubation for 10 min in the dark in 4°C, fluorescence intensity were detected with flow cytometry.

### Colony forming assay

Complete medium containing 1500 single spheroid cells or parent cells was added to 6-well plates and incubated for 10 days. The medium was changed every three days. After discarding the supernatant, the cells were washed for 3 times with PBS, then fixed with 500 μL of methanol for 20 min at room temperature and stained with 0.1% crystal violet (KeyGENE BioTECH, Jiangsu, China) for 20 min. Finally, the colony forming efficacy was counted. This assay was performed in triplicate.

### Chemoresistance assay

Cells were cultured at 2 × 10^3^ cells per well in a 96-well plate for 24 h and then treated with doxorubicin (Dox, Sigma, St. Louis, MO, USA) at different concentrations (0, 2.5, 5, 7.5, 10, 15, 20 ug/ml) for another 24 h, after which 10 μl cell counting kit-8 (CCK-8) reagent (Dojindo, Kumamoto, Japan) was added. Absorbance was detected after incubating for 1.5 h at 450 nm and microplate reader (Tecan, Infnite®M200, Austria) was used. Experiments were performed in 4 replicate wells per sample.

### Wound healing assay and transwell assay

Wound healing assay was used to evaluate the cell migration ability [21].The spheroid and parent cells were seeded in a 12-well plate. A straight line was drawn with the tip of the 10 μl pipette when the density of cells reached 90%. Then cell debris was washed with PBS and the serum-free medium was then added for cell culture. Scrape lines were based on the border of most of the migrating cells and are photographed at different time points (0, 12, 24, 48 h after scrape) using a phase-contrast microscope (Nikon Eclipse Ti-S, Japan). The scratch distance and wound healing rate were calculated using Image J software (National Institutes of Health, Bethesda, MD, USA).

Transwell assay was conducted to detect the migration and invasion ability using transwell chambers (Corning, Shanghai, China) in a 24-well plate [21]. For the invasion assay, the upper chambers were pre-coated with 30 μl matrigel (Corning, Shanghai, China) for 60 min at 37 °C. Spheroid cells and parent cells of Ishikawa and HEC1A were counted and 1 × 10^5^ cells were inoculated in serum-free DMEM medium in a chamber. The lower well was filled with 800 μl DMEM medium containing 20% FBS. After 36 h of culture in a 5% CO_2_ incubator at 37 °C, the upper layer cells were wiped off with a swab. The lower cells of the chamber were fixed with methanol and stained with 0.1% crystal violet (KeyGENE BioTECH, Jiangsu, China). The migration assay was the same as above except that there was no matrigel coated. The results were photographed in five randomized visual fields and the experiments were repeated three times for both assays.

### Xenografted tumor model

The animal experiment was approved by the Animal Center of the Southern Medical University [21] (Additional files 2 and 3). A total of 18 five-week-old BALB/c-nu athymic nude mice were raised in specific-pathogen free (SPF) room(Experimental Animal Center of Southern Medical University, Guangzhou, China) and received a subcutaneous injection in the right flank of cells (5.0 × 10^7^ cells/ 100 μl PBS and 100 μl matrigel). The mice were randomly divided into two groups for inoculation of spheroid and parent cells. Nude mice were injected with spheroid cells and parent cells of Ishikawa (*n* = 3 per group) or HEC1A cells (*n* = 6 per group). Growth curves were plotted using average tumor volume within each experimental group every 3 days. The mice were euthanized on day 21st and 27th using in a CO_2_ container, and the dissected tumors were collected. We declare that our study adheres to the ARRIVE guidelines for the reporting of animal experiments.

### Western blot analysis and antibodies

Western blot was conducted to evaluate the protein expression level. Total proteins were extracted from spheroid cells and parent cells of Ishikawa and HEC1A cells. Radio-immunoprecipitation assay (RIPA) buffer containing 1 mM PMSF (Beyotime, China) was used. Bicinchoninic acid (BCA) protein assay kit (Beyotime, China) was used to detect the protein concentration. The protein sample was mixed with the loading buffer (Beyotime, China) at a volume ratio of 4: 1 and boiled for 10 min to denature it. Then, 30 μg of the mixed sample was separated by 10% sodium dodecyl sulfate -polyacrylamide gel electrophoresis (SDS-PAGE), and then transferred to a polyvinylidene fluoride (PVDF) membrane by a wet transfer machine (Trans-blot SD, BioRad, USA). The membrane was blocked with 5% skim milk for 2 h at room temperature and then incubated with primary antibody at 4 °C overnight. Then the membrane was washed by 1 × Tris Buffered saline Tween (1 × TBST) for 3 times and incubated with corresponding secondary antibody at room temperature for 2 h, followed by washing 3 times with 1 × TBST. Finally, bands were detected using ECL detection Kit and visualized by the SmartChemi610 (Beijing Sage Creation Science, China)[19].

Primary antibodies of western blotting including HK2 (1:1,000, rabbit anti-human, 2A11C3, #66,974–1-Ig; Proteintech Group, INC, USA), GPRC5A (1:1,000, rabbit anti-human, #10,309–1-AP; Proteintech Group, INC, USA), PFKFB3 (1:1,000, rabbit anti-human, #13,763–1-AP; Proteintech Group, INC, USA), and GAPDH (1:5,000, mouse anti-human, conjugated with HRP, 1E6D9, #60,004–1-Ig; Proteintech Group, INC, USA). Secondary antibodies used in the study were goat anti-rabbit IgG conjugated to HRP (1:5000, MultiSciences, China), and goat anti-mouse IgG conjugated to HRP (1:5000, MultiSciences, China).

### Immunohistochemistry (IHC) analysis

Tumors dissected from mice were examined by IHC analysis using a phase-contrast microscope (Nikon Eclipse Ti‑S; Nikon Corp., Japan) [22]. Paraffin-embedded tissues were cut into 5-µm sections for IHC. First, tissues were dehydrated and blocked with 5% goat serum. Then, tissues were incubated with proliferating cell nuclear antigen (PCNA), vascular endothelial growth factor (VEGF), CD133 and hypoxia inducible factor-1α (HIF-1α) (1:200, rabbit anti-human; Proteintech Group, INC, USA) primary antibody at 4˚C overnight. The following step was incubated with corresponding secondary antibody (dilution 1:5,000, Hangzhou MultiSciences Biotech Co., China). Finally, sections were visualized using a diaminobenzidine (DAB) horseradish Peroxidase (HRP) color development kit (Beyotime Institute of Biotechnology, Haimen, China). Image J software was used to determine the staining score by combining the percentage of positive cells and the staining intensity.

### Protein extraction and trypsin digestion

Samples were saved in − 80 °C and were added with 4 times the volume of lysis buffer (8 M urea, 1% protease inhibitor, 1% phosphatase inhibitor), and sonicated[23]. After 12,000 g centrifugation for 10 min at 4 °C, the cellular debris was removed and the supernatant was transferred into a new centrifuge tube. The concentration was detected using the BCA protein assay kit. Protein was added with dithiothreitol (DTT, Sigma, St. Louis, MO, USA) to a final concentration of 5 mM as the reduction at 56 °C for 30 min and iodoacetamide (IAA, Sigma, St. Louis, MO, USA) to a final concentration of 11 mM, and then incubated in the dark at room temperature for 15 min. Finally, the urea (Sigma, St. Louis, MO, USA) concentration of the sample was diluted to less than 2 M. Trypsin (Promega, Madison, WI, USA) was added at the mass ratio of 1:50 (trypsin: protein) at 37 °C for overnight digestion. And then 1: 100 mass ratio for a continuing 4 h digestion.

### Tandem mass tag™ (TMT) labeling and high performance liquid chromatography (HPLC) fractionation

Peptide was digested by trypsin and desalted with Strata X C18 (Phenomenex, California, USA), then vacuum freeze-dried. Triethylamonium bicarbonate (TEAB, 0.5 M, Sigma, St. Louis, MO, USA) was used to dissolve peptide. Dissolved peptides were labeled by the instruction of TMT 6plex kit (Thermo Scientific, Waltham, MA, USA) and the labels of Ishikawa parent cells, Ishikawa spheroid cells, HEC1A parent cells and HEC1A spheroid cells human endometrial cancer cells was 128, 129, 130 and 131. This project has 4 groups, each group contains 3 biological duplicate samples, a total of 12 samples. For each TMT experiment, respective 4 labeled samples were combined and dried in a vacuum centrifuge. In a brief, the labeled reagent was thawed and dissolved in acetonitrile (ACN, Fisher Chemical, Leicestershire, UK) and the peptide mixtures were incubated for two hours. Then the peptide mixtures were pooled, desalted, and vacuum freeze-dried.

Agilent 300 Extend C18 column (5 μm particles, 4.6 mm ID, 250 mm length) was used to fractionate the tryptic peptides by high pH reverse-phase HPLC. The peptides were dissociated into 60 fractions within 60 min at ACN (pH 9.0) of 8% to 32%. Next, the peptides were combined into 9 components and dried using vacuum centrifuging.

### Liquid chromatography-tandem mass spectrometry (LC–MS/MS) analysis

Peptides were dissolved in 0.1% formic acid (FA, solvent A) and separated by EASY-nLC 1000 UPLC system (ReproSil-Pur Basic C18 column size, 1.9 μm,100 μm, 25 cm) [23]. The gradient consisted of increasing solvent B (0.1% FA in 90% ACN) from 6 to 22% over 42 min, 22% to 30% in 12 min, 80% in 3 min then holding at 80% for the last 3 min. The constant flow rate was set at 500 nL/min. The peptide was ionized and analyzed by Orbitrap Fusion Lumos mass spectrometry. The 2.4 kV electrospray voltage was used. For primary MS, m/z scan was 350 to 1,550 with the resolution of 60,000. For secondary MS, the starting scan range was 100 m/z, and the resolution was 15,000. The data acquisition was data dependent scanning (DDA) program. The automatic gain control (AGC) was 5E4 to improve the efficiency of MS. To avoid repeated parent ion scanning, the signal threshold was set to 10,000 ions/s, the maximum injection time was 60 ms and the dynamic exclusion time of tandem mass spectrometry was 30 s.

### Bioinformatic analysis

The secondary mass spectrometry data was retrieved by Maxquant (v1.5.2.8) [24]. Tmt-6plex method was used to quantify and the false discovery rate (FDR) of peptide-spectrum and protein identification was set as 1%. Several bioinformatics tools were used to analyze these data. UniProt-GOA database (http://www.ebi.ac.uk/GOA/) and InterProScan were used to generate Gene Ontology (GO) annotation [25]. InterProScan (http://www.ebi.ac.uk/interpro/) was used to identify proteins domain functions. The Kyoto Encyclopedia of Genes and Genomes (KEGG) database was used to analyze pathways by KEGG Automatic Annotation Server (KAAS) and KEGG mapper. WoLF PSORT was used to predict subcellular location. STRING (v.11.0) protein network interaction database was used to the interaction relationship of differential proteins. Further, cytoscape (v.3.6.0) software was used to visualize the network.

### Small interfering RNA transfection

Small interfering RNA (siRNA) for HK2 (RiboBio, Guangzhou, China) was employed to knock down the expression of HK2 in Ishikawa and HEC1A cells as described previously [22]. The specific siRNA sequences were detailed in Table 2. siRNA was transfected with Lipofectamine 3000 transfection reagent (InvitrogenLife Technologies) according to the manufacturer's instructions. Total RNAs or proteins were isolated 48 h or 72 h after transfection to detect HK2 mRNA and protein expression levels.

**Table 2:** Details of siRNA sequences

Specific siRNA or NC	Sequences
HK2-siRNA-1	CTGTGAAGTTGGCCTCATT
HK2-siRNA-2	ACGACAGCATCATTGTTAA
HK2-siRNA-3	CTGGCTAACTTCATGGATA
NC	UUCUCCGAACGUGUCACGUTT

### RNA-seq analysis

Total RNA extraction in four groups (Ishikawa-NC V.S Ishikawa-HK2-siRNA, HEC1A-NC V.S HEC1A-HK2-siRNA) was used by Trizol for library preparation, and the sequencing platform was Illumina sequencing. Sequencing and analysis of transcriptome of the samples were performed in Sangon Biotech Co., Ltd (Shanghai, China). Use DESeq2 software (1.16.1) to perform differential expression analysis between two comparative combinations (Ishikawa-NC V.S. Ishikawa-HK2-siRNA, HEC1A-NC V.S. HEC1A-HK2-siRNA). Then the overlapping genes of Ishikawa and HEC1A were proceeded KEGG enrichment analysis (Kanehisa and Goto 2000) [26].

### Statistical analysis

Data are expressed as mean ± standard deviation (mean ± SD). The Student’s* t* test was used for comparisons between two groups (SPSS 22.0 software, IL, USA). *P* < 0.05 was considered as statistically significant. Fold change (FC) > 1.5 or FC < 0.667 compared to parent cell group were considered as significantly dysregulated for identified proteins.

## Results

### The ability of colony forming, subsphere-forming and drug resistance of spheroid cells

**Fig. 1:**
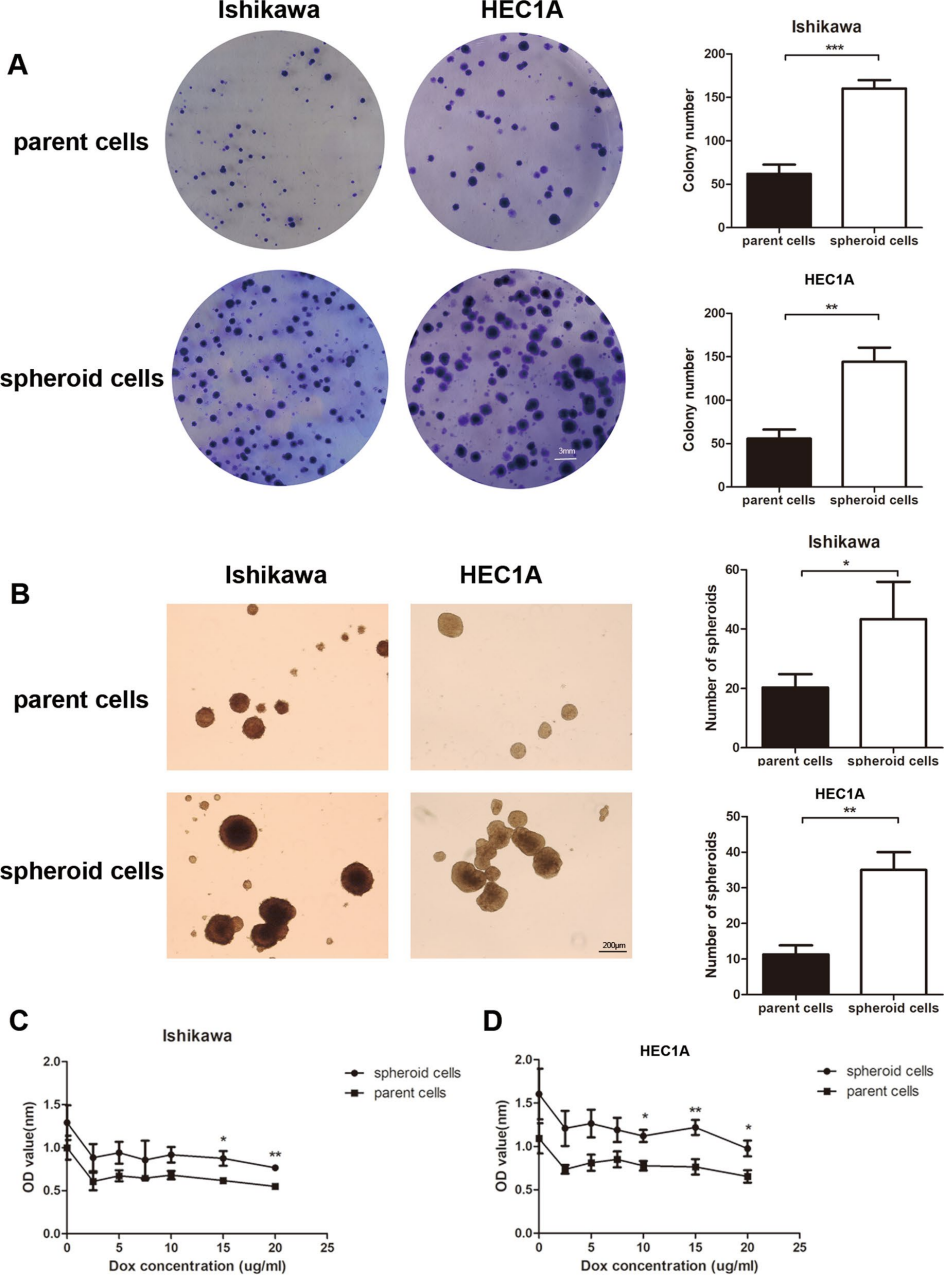
Spheroid cells showed increased ability of colony forming, subsphere forming and drug resistance. **A** Colony forming assay and quantitative efficiency of the parent cells and spheroid cells. **B** Secondary spheroid body formation assay and number of secondary spheroids of the parent cells and spheroid cells. **C** Rates of resistance to dox were assessed using CCK8 assay for Ishikawa. **D** Rates of resistance to dox were assessed using CCK8 assay for HEC1A (* *P* < 0.05, ** *P* < 0.01, *** *P* < 0.001)

Spheroid cells showed higher capacity of colony formation than parent cells on the sixth day of culture (Fig. 1A). The subsphere-forming assay suggested that spheroid cells cultivated in serum-free medium were more likely to form secondary spheres than parent cells (*P* < 0.05 in Ishikawa cells, *P* < 0.01 in HEC1A cells) which represented the ability of self-renewal (Fig. 1B). Moreover, the capacity of resistance to Dox in vitro were also assessed. Spheroid cells had higher rates of survival under the treatment of 15 and 20 µg/ml Dox in Ishikawa cells (*P* < 0.05 and *P* < 0.01, respectively) and 10, 15 and 20 µg/ml Dox in HEC1A cells (*P* < 0.05, *P* < 0.01, and* P* < 0.05, respectively) (Fig. 1C and D). All the assays were performed in both Ishikawa cells and HEC1A cells.

### The ability of migration and invasion of spheroid cells

**Fig. 2:**
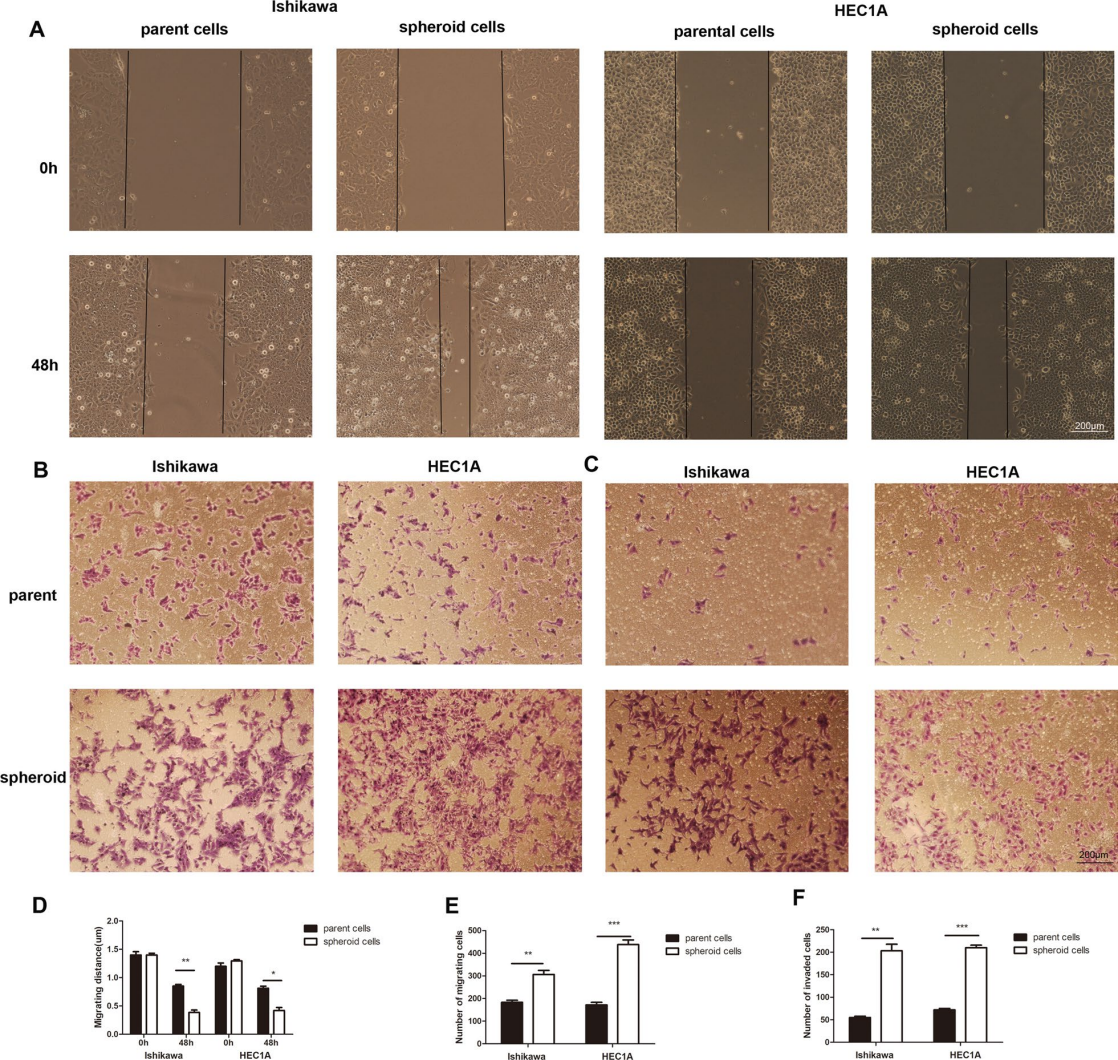
Spheroid cells showed enhanced ability of migration and invasion. **A** Wound healing assay was used to assess the migration ability of parent cells and spheroid cells in Ishikawa and HEC1A at 48 h. **B**, **C** The invasion of parent cell and spheroid cells in Ishikawa and HEC1A. **D**, **E**, and **F** shows data from three independent experiments from **A**, **B**, and **C** respectively. Scale bar, 200 μm. Data are expressed as mean ± SD (* *P* < 0.05, ** *P* < 0.01, *** *P* < 0.001)

We observed from the wound healing assays that spheroid cells had enhanced migration rate compared with parent cells in Ishikawa and HEC1A cell lines (*P* < 0.01 and *P* < 0.05, respectively) (Fig. 2A and D). Transwell assays exhibited that spheroid cells had increased migration (*P* < 0.01 and *P* < 0.001) (Fig. 2B and E) and invasion ability (*P* < 0.01 and *P* < 0.001) (Fig. 2C and F).

### Spheroid cells originated from Ishikawa and HEC1A expressing the stem cell markers

**Fig. 3:**
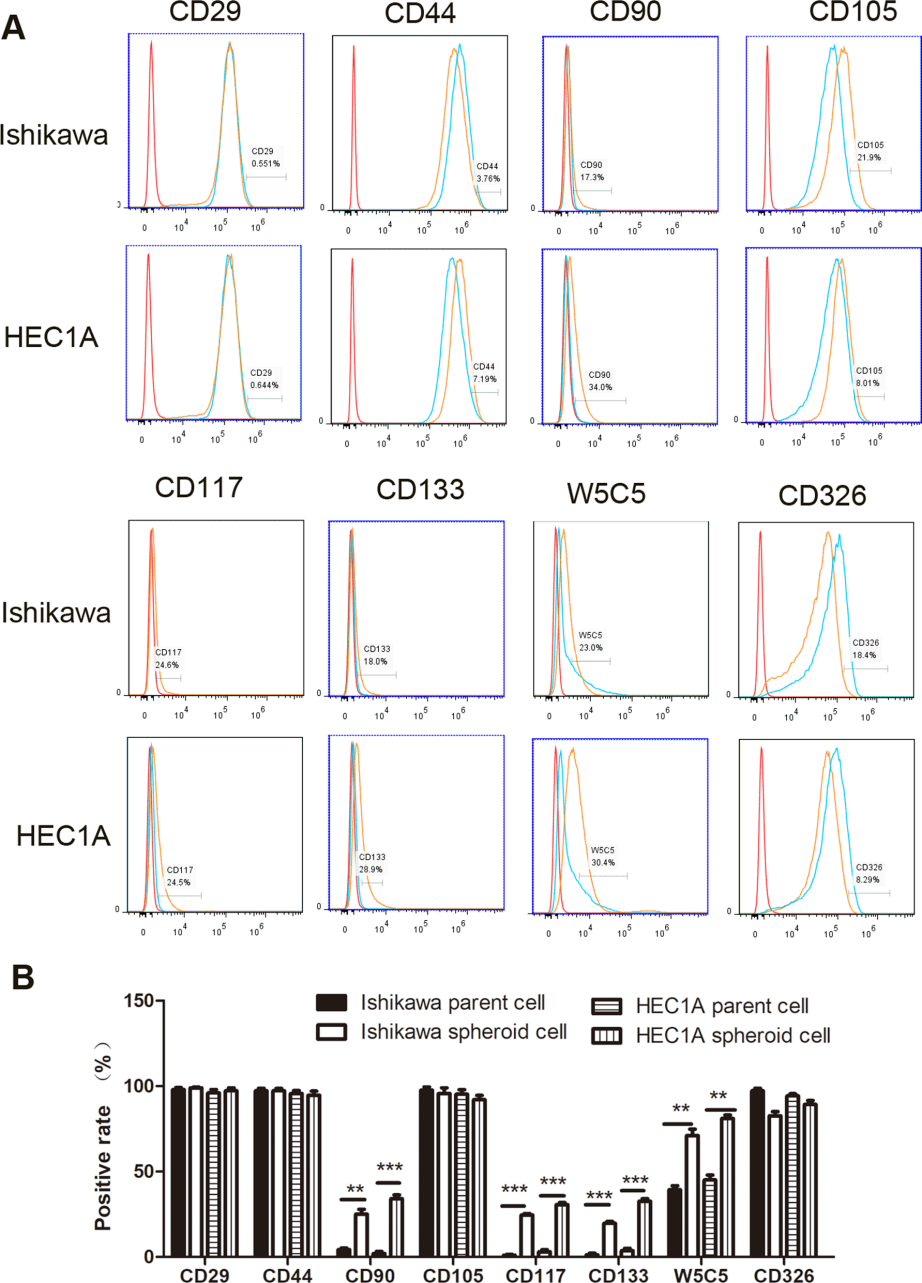
Spheroid cells originated from Ishikawa and HEC1A expressed the stem cell related markers. **A** The expression patterns of a series of stem cell related marker CD29, CD44, CD90, CD105, CD117, CD133, W5C5 and CD326 were analyzed by flow cytometry in human endometrial cancer cell lines Ishikawa and HEC1A and their spheroid cells using serum-free suspension culture method, respectively. Red lines represent control group, blue lines represent parent cell and orange ones represent spheroid. **B** Proportions of cells expression stem cell related markers were quantitatively demonstrated in Ishikawa spheroid cells and HEC1A spheroid cells. Data in bar graphs displayed as mean ± SD (** *P* < 0.01, *** *P* < 0.001)

We used flow cytometry to examine that spheroid cells from Ishikawa and HEC1A express the markers of stem cells, including the mesenchymal cell markers CD29, CD44, CD90, CD105, CD117, CD133 and W5C5, and epithelial cell markers CD326 (Fig. 3A). As expected, spheroid cells showed increased expression of CD90, CD117, CD133 and W5C5. The positive rates of the above-mentioned stem cell-related markers are 17.3%, 24.6%, 18.0% and 23.0% higher for spheroid cells of Ishikawa and 34.0%, 24.5%, 28.9% and 30.4% higher for spheroid cells of HEC1A (Fig. 3B) compared with their parent cells, respectively. However, there was no differences for other stem cell markers, including CD29, CD44, CD105 and epithelial cell markers CD326 between spheroid cells and parent cells.

### Spheroid cells showed higher tumorigenicity in vivo

**Fig. 4:**
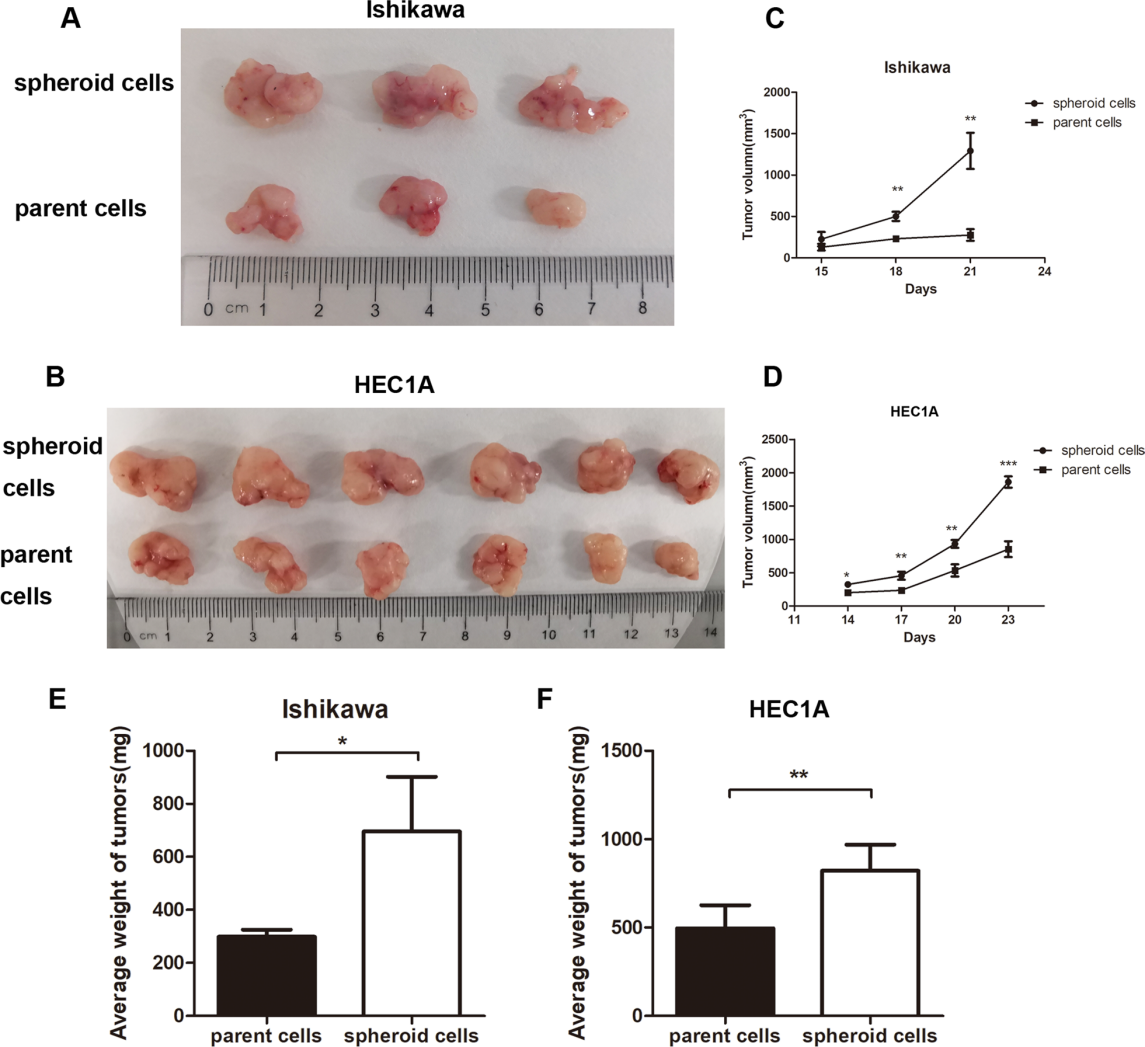
Spheroid cells showed improved tumor forming ability in the in vivo xenograft study. **A**, **B** Xenograft tumor growth of nude mice injected with spheroid cells and parent cells of Ishikawa (*n* = 3 per group, A) or HEC1A cells (*n* = 6 per group, B). The volume **C, D** and weight **E**, **F** of xenografted tumors transfected with Ishikawa parent cells and spheroid cells were measured and analyzed, respectively. Tumor volume was calculated every three days after injection, and the tumor was excised and weighed after 21 days and 23 days. Bar graphs display mean ± SD (* *P* < 0.05 vs. parent cells, ** P < 0.01 vs. parent cells, *** *P* < 0.001 vs. parent cells)

**Fig. 5:**
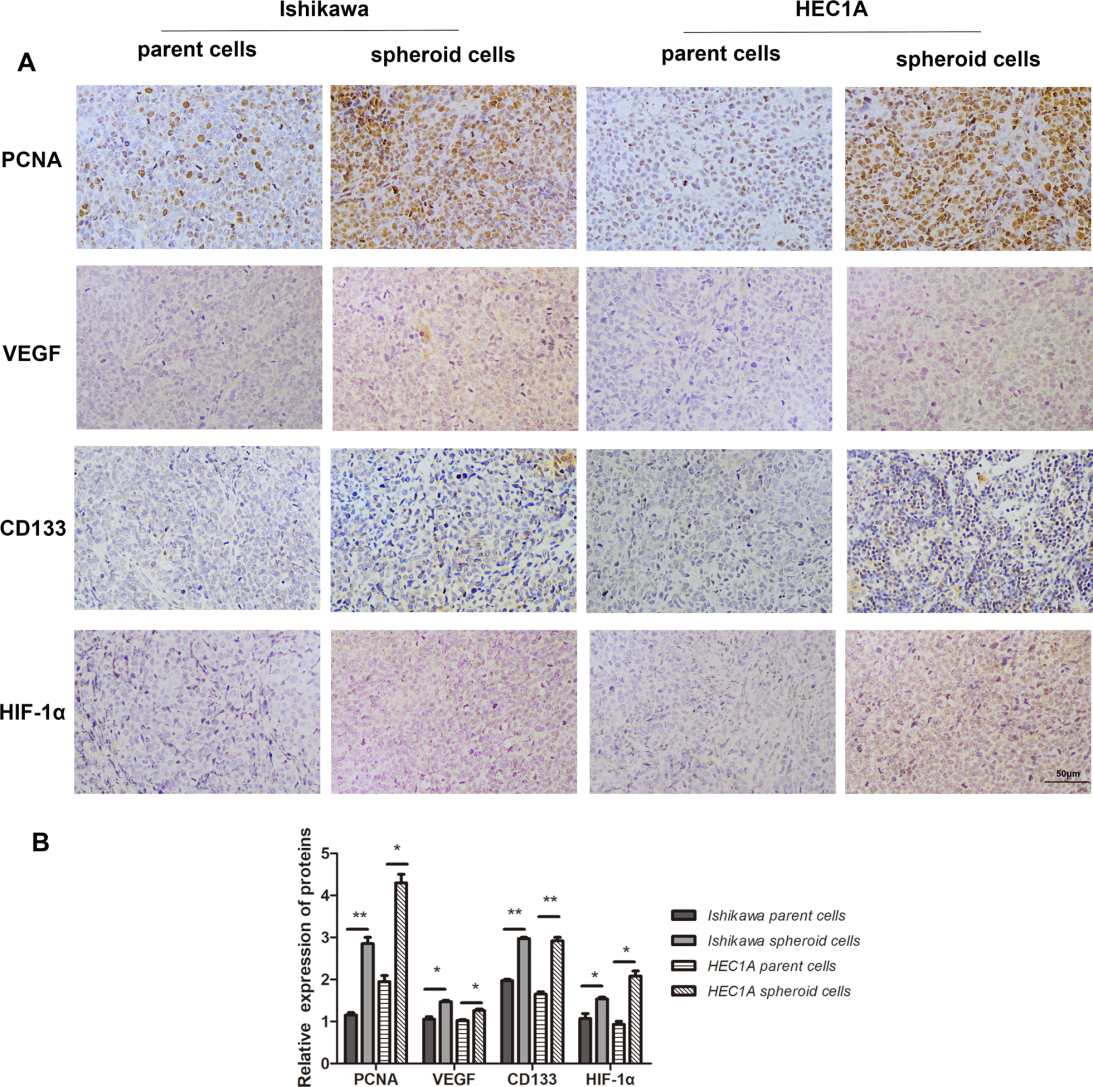
IHC results of xenograft tumors transfected with spheroid cells and parent cells of Ishikawa and HEC1A, respectively. **A** PCNA, VEGF, CD133 and HIF-1α were analyzed by IHC. **B** The positive staining of PCNA, VEGF, CD133 and HIF-1α were calculated by Image J software. Scale bar, 50 μm. Bar graphs display mean ± SD (* *P* < 0.05 vs. parent cells, ** P < 0.01 vs. parent cells)

The typical photos of tumors were showed in Fig. 4A–B. Mice injected with spheroid cells generated tumors in enhanced volume (*P* < 0.01) and weight (*P* < 0.05 in Ishikawa cells, *P* < 0.01 in HEC1A cells) (Fig. 4C–F). Besides, spheroid cells-generated tumors showed the cellular robust activity compared with parent cells tumor as proliferative-related protein PCNA, angiogenesis-related protein VEGF, CSC-related protein CD133 and HIF-1α displayed significantly increased expressions in spheroid cells induced tumors (Fig. 5A–B).

### Analysis of differentially expressed proteins by TMT-based quantitative proteomics

**Fig. 6:**
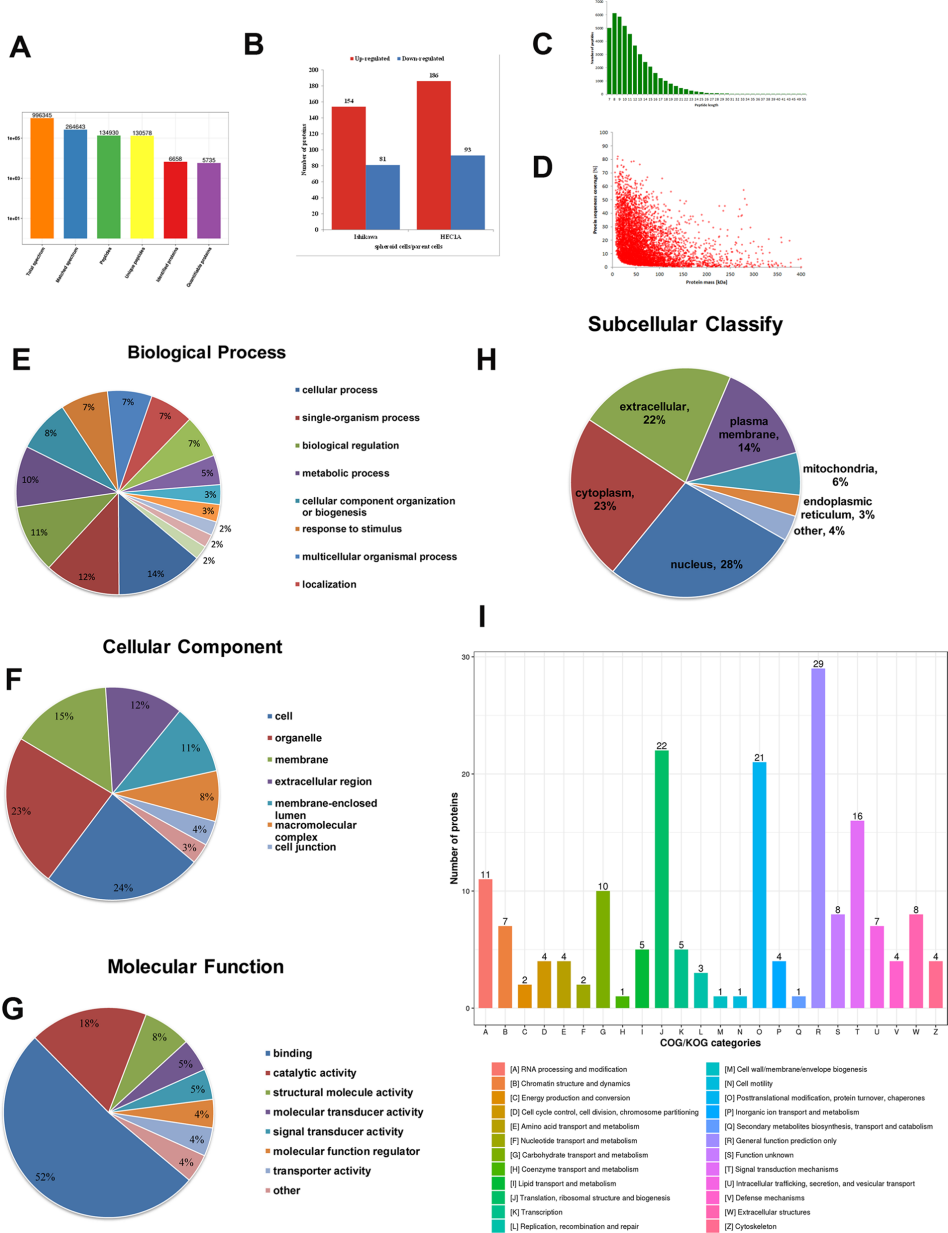
Results of proteomic quality control and Gene Ontology (GO) analysis of differentially expressed proteins in spheroid cells comparing to parent cell in Ishikawa and HEC1A. **A** Basic statistics of mass spectrometry data. **B** Number of differentially expressed proteins between spheroid and parent cells in Ishikawa and HEC1A. **C** Identification of the length distribution of the peptides by mass spectrometry. **D** Identification of the relationship between protein molecular weight and coverage by mass spectrometry. **E** Biological process, **F** cellular component, and **G** molecular function analysis. **H** Subcellular localization and classification of differentially expressed proteins. **I** COG/KOG classification of differentially expressed proteins

Proteomics analysis were performed using protein extractions from parent and spheroid cells from endometrial cancer cell lines. A total of 996,345.0 spectrum were obtained by mass spectrometry analysis. The matched spectrum of the mass spectrum was searched for protein theoretical data, and the number of available valid spectra was 264,643.0, and the spectrum utilization rate was 26.6%. A total of 134,930.0 peptides were identified through spectrum analysis, of which the unique peptides reached 130,578. We identified a total of 6658 proteins, of which 5735 were quantifiable (Fig. 6A). The length distribution of the peptides and the relation of protein molecular weight and coverage was showed in Fig. 6C and D. We used > 1.5 or < 0.67 as the cut-off ratio for differentially expressed protein identification and *P* < 0.05 was considered significant. 154 and 186 proteins were up-regulated and 81 and 93 were down-regulated in Ishikawa and HEC1A cells, respectively (showed in Fig. 6B).

### Functional enrichment analysis of spheroid cells comparing to parent cells of endometrial cancer

**Fig. 7:**
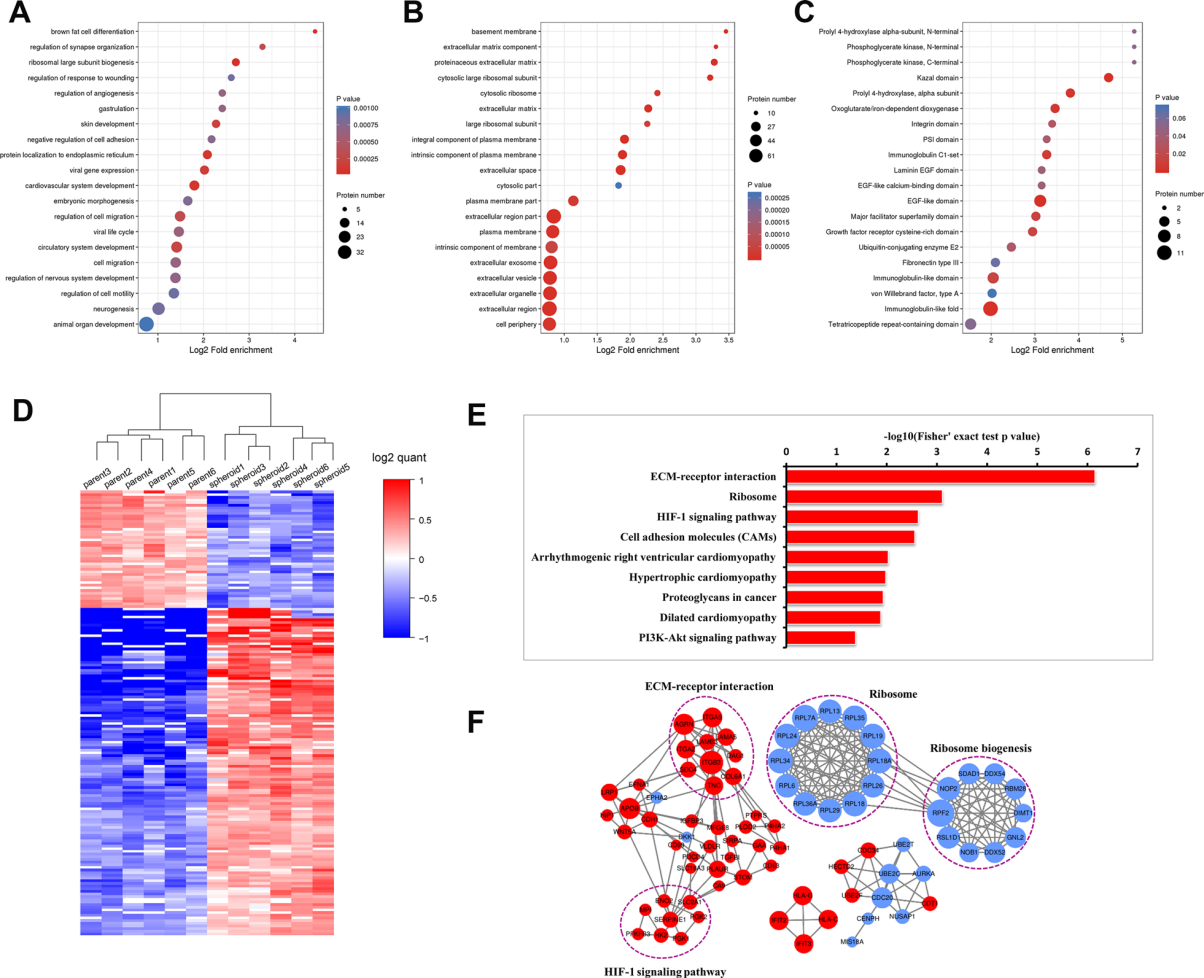
Results of proteomic analysis of differentially expressed proteins. Bubble charts of **A** Biological process; **B** Cellular component; **C** Molecular function. Red represents high expression, while blue represents low expression. Significant enrichment (*P* < 0.05) of differentially expressed proteins was revealed by Fisher's exact test and the top 20 most significantly enriched categories are shown in the bubble chart. The vertical axis of the bubble chart is the functional classification or pathway, and the horizontal axis is the Log2 converted value of the proportion of the differentially expressed protein in the functional type divided by the ratio of the identified proteins. The circle color indicates the enrichment *P* value, and the circle size indicates the number of differentially expressed proteins in the functional classes or pathways. **D** Clustering analysis was performed to display the significant differentially expressed proteins in spheroid cells and parent cells of Ishikawa and HEC1A. Red represents higher expression, while blue represents lower expression (Ishikawa: parent 1–3 and spheroid 1–3, HEC1A: parent 4–6 and spheroid 4–6). **E** KEGG pathway enrichment in spheroid cells and parent cells of Ishikawa and HEC1A. **F** STRING analysis revealed the protein–protein interaction (PPI) network of the 80 dysregulated proteins. Circles represent differentially expressed proteins, and different colors represent differentially expressed proteins (blue is down-regulated protein, red is up-regulated protein). The circle size represents the number of proteins that are different from each other. The larger the circle, the more proteins it interacts with, the more important the protein is in the network

GO classification (showed in Fig. 6E–I), KEGG pathway and protein domain analysis were performed for differentially expressed proteins in order to discover the significant enrichment trends in specific functional types, and the final results were the consistent part of the Ishikawa and HEC1A groups and were shown in the form of bubble charts. *P* values were calculated by the Fisher's exact test. The vertical axis of the bubble chart contained the functional classification or pathway, and the horizontal axis is the Log2 converted value of the percentage of differential proteins in this functional type divided by the proportion of identified proteins. The result of biological process showed that the differentially expressed proteins are mainly enriched in brown fat cell differentiation (Fig. 7A). The result of cellular component showed that the differentially expressed proteins are associated with extracellular and plasma membrane component (Fig. 7B). The result of molecular function showed that the differentially expressed proteins are mainly enriched in dioxygenase activity (Fig. 7C) [27].

For a total of 167 overlap differentially expressed proteins of the two cell lines, 43 were up-regulated and 124 were down-regulated. Clustering analysis displayed the significant differentially expressed proteins in spheroid cells and parent cells of Ishikawa and HEC1A (Fig. 7D). The results of KEGG analysis showed that the genes of the differentially expressed proteins primarily associated with HIF-1 signaling pathway, ECM-receptor interaction, PI3K-Akt signaling and Glycolysis/ Gluconeogenesis (Fig. 7E) [27].

### Interaction network analysis of spheroid cells comparing to parent cells of endometrial cancer

To clearly demonstrate the interactive relationship between proteins and identify core regulatory genes, we selected the top 50 proteins with the closest interaction relationship and mapped the protein–protein interaction (PPI) network (Fig. 7F). STRING (v.11.0) was used to analyze the interactions of differentially expressed proteins. KEGG pathway enrichment analysis and PPI network suggest that HIF-1 pathway is essential in spheroid cells, and several researches have indicated that HIF-1 signaling pathway is closely connected with CSCs [28, 29].

### Quantitative analysis of selected proteins in spheroid cells comparing to parent cells of endometrial cancer

**Fig. 8:**
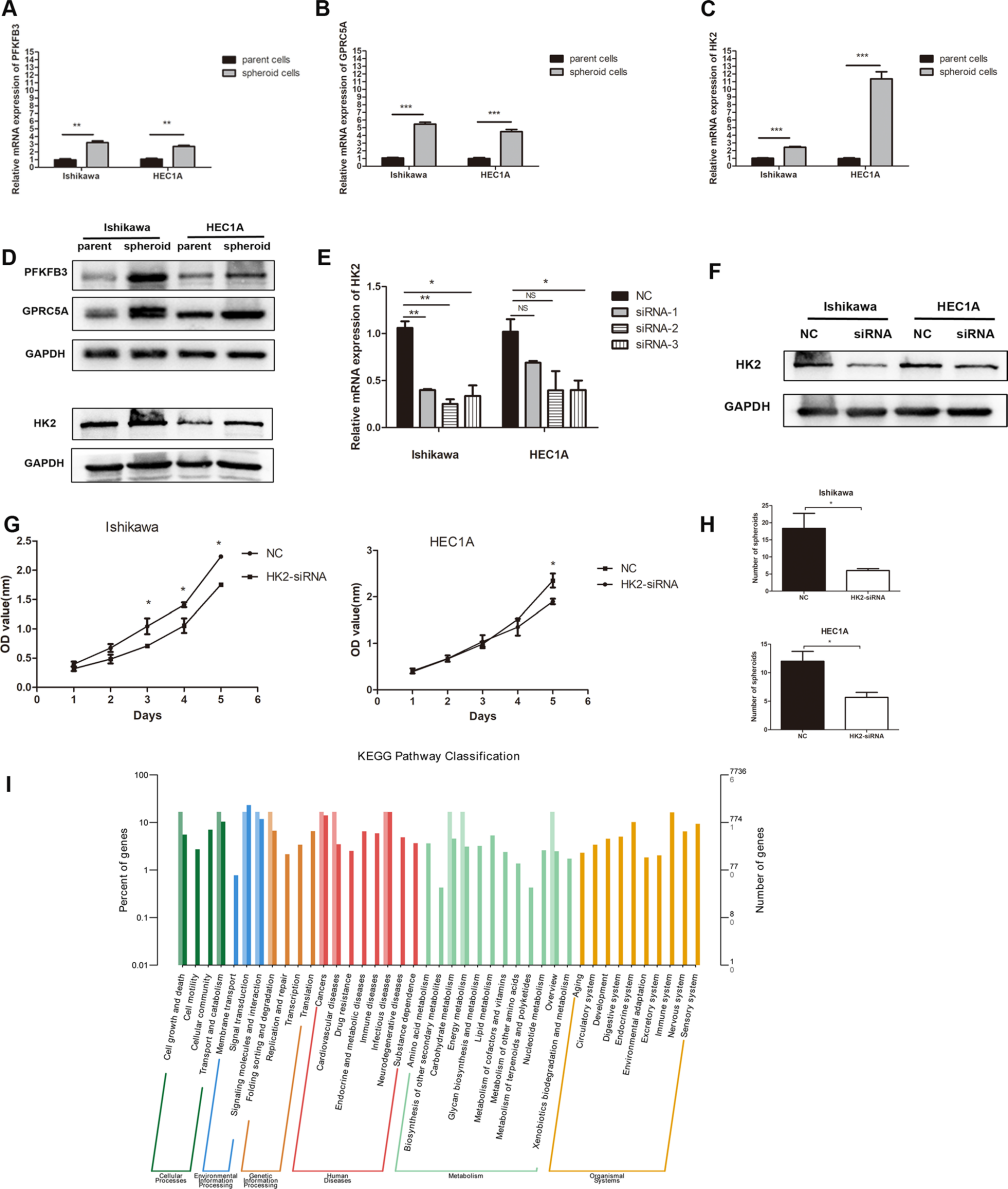
Functional assays of selected targets, PFKFB3, GPRC5A and HK2. **A**–**C** The mRNA levels of PFKFB3, GPRC5A and HK2 were detected by qRT-PCR. **D** The expression levels of PFKFB3, GPRC5A and HK2 were detected by Western blot (Full-length blots are presented in supplementary material). **E** qRT-PCR results for Ishikawa and HEC1A transfected with HK2-siRNA-1, HK2-siRNA-2, HK2-siRNA-3 and NC. **F** Western blot analysis revealed significant downregulation in HK2-siRNA cells in comparison with the controls (Full-length blots are presented in supplementary material 1 and 2). **G** CCK-8 assays for Ishikawa and HEC1A cells transfected with HK2-siRNA or NC. **H** The number of spheroids for Ishikawa and HEC1A transfected with HK2-siRNA or NC in spheroid formation assay. **I** KEGG biochemical classification for HK2-siRNA endometrial cancer cells (*P < 0.05, **P < 0.01, *** *P* < 0.001)

Among the 6 differentially expressed proteins in HIF-1 signaling pathway, we selected 3 for validation by qRT-PCR and western blot. The results are consistent with the proteomics analysis results. qRT-PCR showed that the expression levels of 6-phosphofructo-2-kinase/fructose-2,6-bisphosphatase 3 (PFKFB3), G protein coupled receptor, family C, group 5, member A (GPRC5A), and Hexokinase 2 (HK2) were augmented (Fig. 8A–C). Boosted expression of GPRC5A, PFKFB3 and HK2 at protein levels were verified with western blot (Fig. 8D and Additional file 1).

### HK2 knockdown reduced the proliferation of endometrial cancer cells

Endometrial cancer cell lines Ishikawa and HEC1A were transfected with HK2-siRNA (HK2-siRNA-1, HK2-siRNA-2 and HK2-siRNA-3) or negative control. The results of qRT-PCR revealed that the relative mRNA expression levels of HK2 were significantly downregulated in HK2-siRNA-3 transfected cells (Fig. 8E). HK2-siRNA-3 was selected for the following experiments. Western blot was performed to detect the protein levels of HK2, which was consistent results (Fig. 8F  and Additional file 1).

CCK8 assay was performed to explore the effect of HK2 on the proliferation of endometrial cancer cells. The HK2-siRNA cells showed significantly decreased cell viability compared with the HK2-NC group in Ishikawa and HEC1A (Fig. 8G). The spheroid formation assay showed that knock-down HK2 in Ishikawa and HEC1A cells impaired their ability of self-renewal (Fig. 8H). RNA-seq was performed to explore the molecular mechanism of how HK2 promote stemness. There were 54 overlapped and significantly different genes between Ishikawa and HEC1A cells. The pathways had 40 clades under six major KEGG categories, including ‘cellular processes’, ‘environmental information processing’, ‘genetic information processing’, ‘human diseases’, ‘metabolism’, and ‘organismal systems’ (Fig. 8I).

## Discussion

Our experiments evaluated the ability of colony forming, subsphere forming, drug resistance, migration and invasion between spheroid cells and parent cells from endometrial cancer and found that spheroid cells possess greater capacity in the above assays. The xenografted tumor model also proved higher tumorigenicity of spheroid cells. We examined the expression of several surface markers and verified the high expression of CD133 in spheroid cells, which was consistent with previous reports of ECSCs. Moreover, surface markers for other cancer stem cells were also highly expressed in endometrial spheroid cells. Furthermore, the differential proteomic analysis results shed some light on the potential regulator of ECSCs, including HIF-1 pathway, PFKFB3, GPRC5A and HK2.

The recurrence and metastatic features of endometrial cancer is an intractable clinical problem. However, endometrial cancer often reacts poorly to chemo- and radio-therapy, and one of the responsible reasons is the presence of CSCs [30]. Hubbard et al. has provided one of the first evidences for the existence of ECSCs [11]. They identified a small population of endometrial cancer cells which are clonogenic, and can initiate and differentiate into tumors that are similar to parental tumor cells in the xenotransplantation model. CSCs are involved in drug-resistance, progression, invasiveness and metastasis of tumor [31]. Previous studies in endometrial cancer have provided some preliminary data suggesting that spheroid forming assay was an effective way for isolating the ECSCs [12]. Our study also verified that spheroid cells possess cancer stem cells characteristics, including colony forming, subsphere forming, drug resistance, migration, invasion ability and tumorigenicity. Expression of a series of surface markers were verified in this study. Expression of CD90, CD117, CD133 and W5C5 were enhanced in spheroid cells, whereas CD29, CD44 and CD105 were not uniquely expressed in spheroid cells. CD133 were confirmed to be a putative surface for several cancer stem cells, including endometrial cancer [17]. CD117 has been identified as a CSC marker for ovarian cancer and lung cancer [32, 33]. W5C5 was a potential progenitor cell marker of soft-tissue sarcoma, and also considered as a surface marker of normal endometrial mesenchymal stem cells [34, 35]. CD326 is considered as a cancer stem cell-derived biomarker and has been used in breast cancer [36, 37]. However, CD326 was not uniquely expressed in spheroid cells from endometrial cancer.

Furthermore, we performed TMT-based quantitative proteomic analysis of spheroid cells and parent cells in Ishikawa and HEC1A cells to explore more markers and potential regulator of CSCs. Differential proteomics is one of the subtypes of proteomics focusing on identifying the differences between samples in different states or species at protein level. It is of broad use for screening tumor biomarkers, drug mechanism, detection of disease progression and efficacy observation. KEGG is an information network connecting known molecular interactions, such as metabolic pathways, complexes, and biochemical reactions. KEGG pathway mainly includes: metabolism, genetic information processing, environmental information processing, cellular process, human disease, and drug development. Hopefully, it may eventually help find potential therapeutic targets for endometrial cancer and predict the prognosis.

The proteomic results showed that the expression of proteins in spheroid cells differed significantly from parent cells. KEGG pathway analysis indicated that HIF-1 pathway was notably activated in spheroid cells. Hypoxia plays critical role of tumorigenesis and poor prognosis of many solid tumors, such as glioblastoma, prostate cancer as well as endometrial cancer [38–40]. CSCs tend to reside in low oxygen regions because hypoxic tension contributes to initiate and maintain the stemness, and thus hypoxia provides the microenvironment where tumor cells can survive and thrive [41, 42]. HIF-1α only exists in low oxygen condition and leads to a series of adaptive responses by reducing oxygen utilization and producing relevant stimulators. It is considered to be the strongest inducer of glycolytic enzymes, VEGF and erythropoietin and many CSC-signature genes such as CD44 and CD133, Oct-4, Sox-2, Nanog and Myc [43, 44]. Besides, HIF-1α promotes the EMT and the following enrichment of stem-like side population cells in prostate cancer [40]. Interaction between HIF-1 and Notch pathway in a variety of cancer cell lines, is rather important for stem cell maintenance under hypoxia [43–46]. Additionally, a research revealed that hypoxia stimulated HIF-1α and HIF-2α-dependent expression of AlkB homolog 5, which can demethylate Nanog mRNA and increase breast cancer stem cells [47]. HIF-1α mainly controls acute hypoxia-induced cell invasion, while HIF-2α controls chronic hypoxia-induced sphere formation [38]. The sophisticated function of HIF-1 pathway in endometrial cancer still required further research to confirm.

Validation experiments confirmed the increased expression of HK2, PFKFB3 and GPRC5A in spheroid cells, which was consistent with the proteomics results. HK2 and PFKFB3 are key molecules in HIF-1 pathway. HK2 phosphorylate glucose to produce glucose-6-phosphate, which is the first step in most glucose metabolism pathways. Moreover, the HectH9/HK2 pathway regulates CSC expansion and CSC-associated chemoresistance [48]. HK2 knock down in endometrial cancer cells impaired cell proliferation and self-renewal ability, and the RNA-seq of HK2-siRNA cells indicated that HK2 may promoted cancer stemness by metabolic pathways. PFKFB3 is an essential regulator of endothelial glycolysis metabolism, promotes angiogenesis, and thus promote metastasis in malignant tumors [49, 50]. PFKFB3 is quite uniquely found in CSCs, which help discriminate CSCs from non-stem cells and induced pluripotent stem cells (iPS) especially under hypoxic conditions [51]. PFKFB3 inhibitor in endothelial cells can improve vascular maturation and perfusion, restrain tumor invasion, intravasation and metastasis, and thus can be considered as a promising therapeutic target [52]. It has been reported that GPRC5A promote the self-renewal and metastasis of bladder CSCs [53, 54]. Mutation of GPRC5A promote cell differentiation and self-renewal pathway and significantly increased the sphere-forming ability of bladder cancer non-stem cells [54]. In lung tumor, GPRC5A deficiency leads to murine double minute 2 overexpression, which is a p53 negative regulator, and promotes tumorigenicity of the tumor cells [55]. However, roles of above-mentioned proteins in endometrial cancer have not been reported and still need further exploration.

## Conclusions

Taken together, we verified that spheroid cells of endometrial cancer possessed stem cell-like characteristics and enriched CSCs. Then we conducted the TMT-based quantitative proteomic analysis of spheroid cells in endometrial cancer and found that HIF-1 pathway was activated and might involve in regulation of CSCs. We subsequently confirmed that HK2, PFKFB3 and GPRC5A were over-expressed in spheroid cells and could be potential ECSCs markers and regulation targets. Methods aiming at reducing CSC-containing spheres may diminish the invasiveness of tumors. In clinical studies, the identification of specific markers of ECSC is also essential for prognosis prediction, clinical classification and drug development. Our study might support that HIF-1 inhibition could be prospective treatment option in the future.

## Supplementary Information

**Additional file 1**. Original uncropped blots of the WB figure in the combined picture.

**Additional file 2**. Ethical approval 1.

**Additional file 3**. Ethical approval 2.

## Data Availability

The mass spectrometry proteomics data have been deposited to the iProX with the dataset identified IPX0003686000; https://www.iprox.cn/page/SSV024.html;url=16788010467287doD; password: hQGP. The gene expression data of RNA-seq data of knock-down HK2 in Ishikawa and HEC1A cells are deposited to the GEO with the dataset identified GSE222599. Other data used during this study are available from the corresponding author on reasonable request.

## Abbreviations

ACN: Acetonitrile
AGC: Automatic gain control
BCA: Bicinchoninic acid
CCK-8: Cell counting kit-8
CSCs: Cancer stem cells
DAB: Diaminobenzidine
DDA: Data dependent acquisition
Dox: Doxorubicin
DTT: Dithiothreitol
EC: Endometrial cancer
EMT: Epithelial-mesenchymal transition
ESCS: Endometrial cancer stem cells
FA: Formic acid
FACS: Fluorescence activated cells sorting
FBS: Fetal bovine serum
FC: Fold change
FDR: False discovery rate
GO: Gene ontology
GPRC5A: G protein coupled receptor: family C: group 5: member A
HIF-1α: Hypoxia inducible factor-1α
HK2: Hexokinase 2
HPLC: High performance liquid chromatography
HRP: Horseradish peroxidase
IHC: Immunohistochemistry
KEGG: The Kyoto Encyclopedia of genes and genomes
LC–MS/MS: Liquid chromatography-tandem mass spectrometry
MACS: Magnetic activated cells sorting
PCNA: Proliferating cell nuclear antigen
PFKFB3: 6-Phosphofructo-2-kinase/fructose-2,6-bisphosphatase 3
PPI: Protein–protein interaction
PVDF: Polyvinylidene fluoride
qRT-PCR: Quantitative RT-PCR
RIPA: Radio-immunoprecipitation assay
SDS-PAGE: Sodium dodecyl sulfate -polyacrylamide gel electrophoresis
SP: Side population
TBST: Tris buffered saline tween
TEAB: Triethylamonium bicarbonate
TMT: Tandem mass tag™
V: Volume
VEGF: Vascular endothelial growth factor
