# Supplementary figures and images for: TMT-based quantitative proteomic analysis of spheroid cells of endometrial cancer possessing cancer stem cell properties
Source: Stem Cell Res Ther. 2023 May 4;14:119. doi: 10.1186/s13287-023-03348-x (PMC10161517; doi:10.1186/s13287-023-03348-x)

1. Original images of Figure 8D


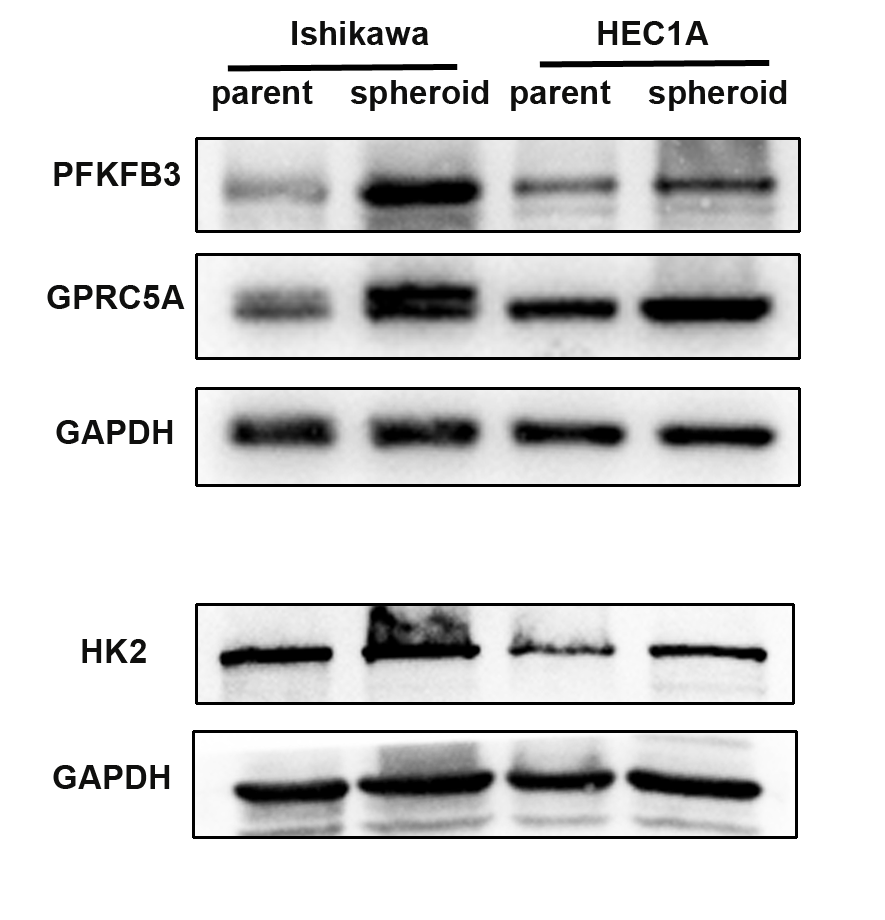


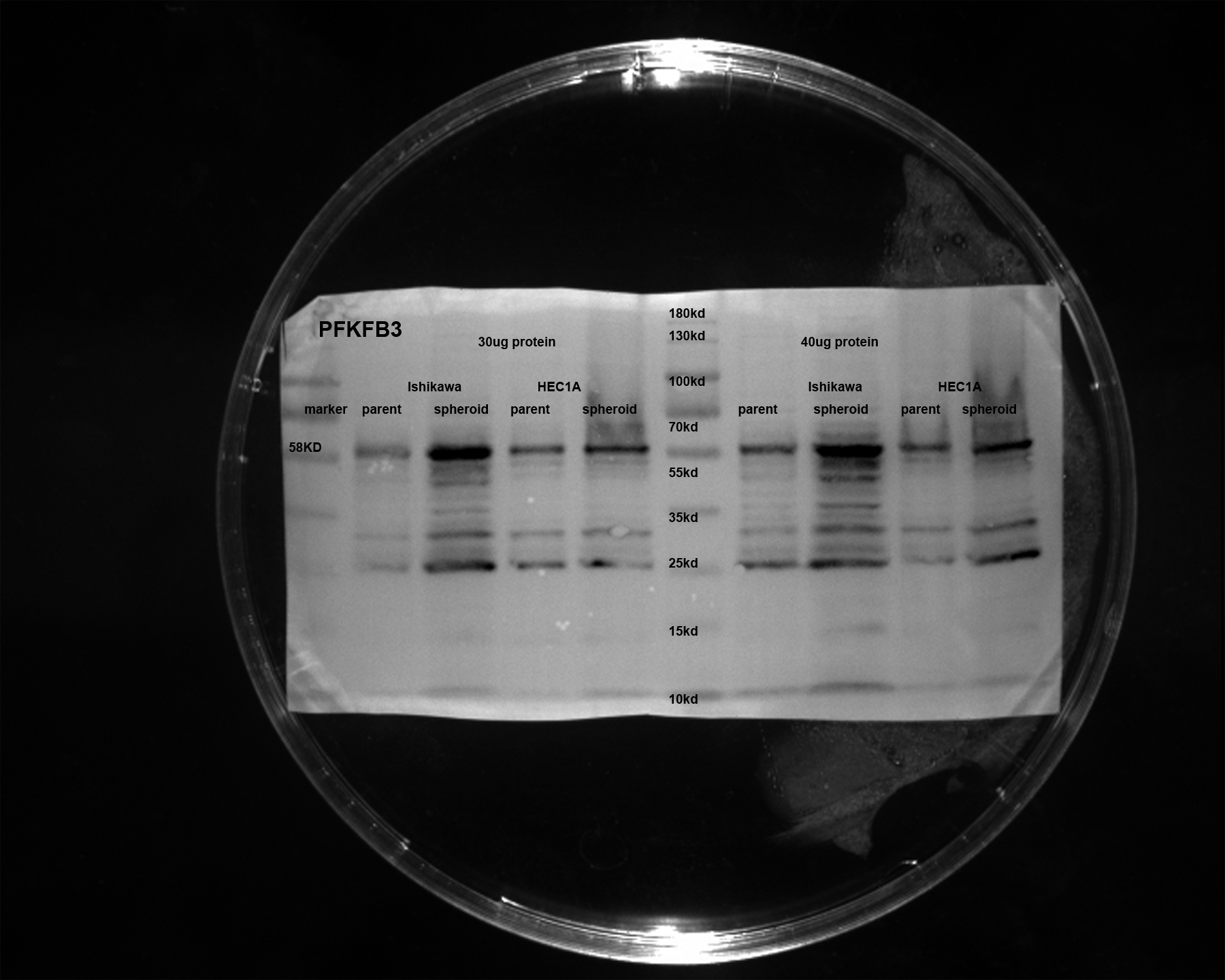


Sfig1-1


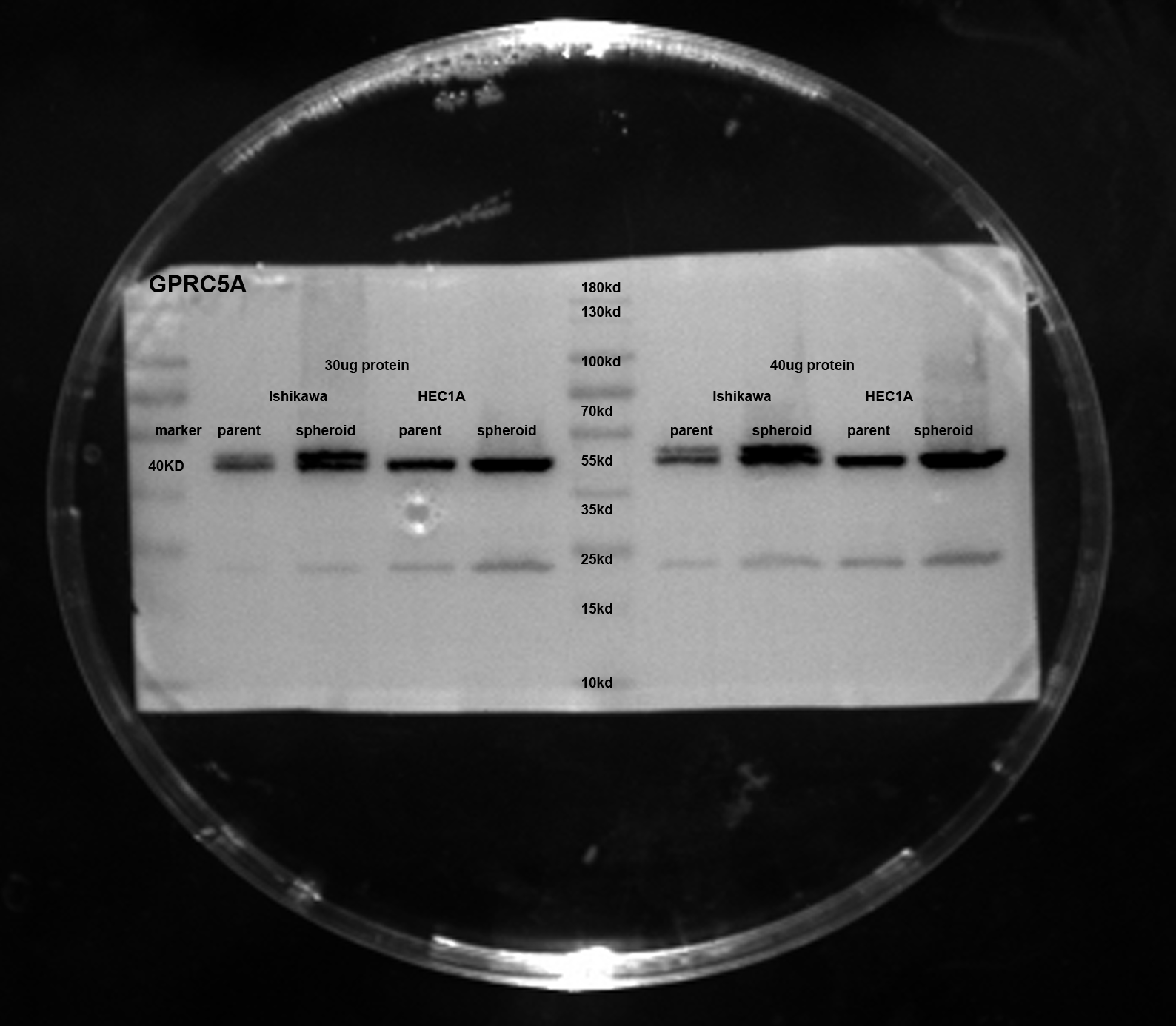


Sfig1-2


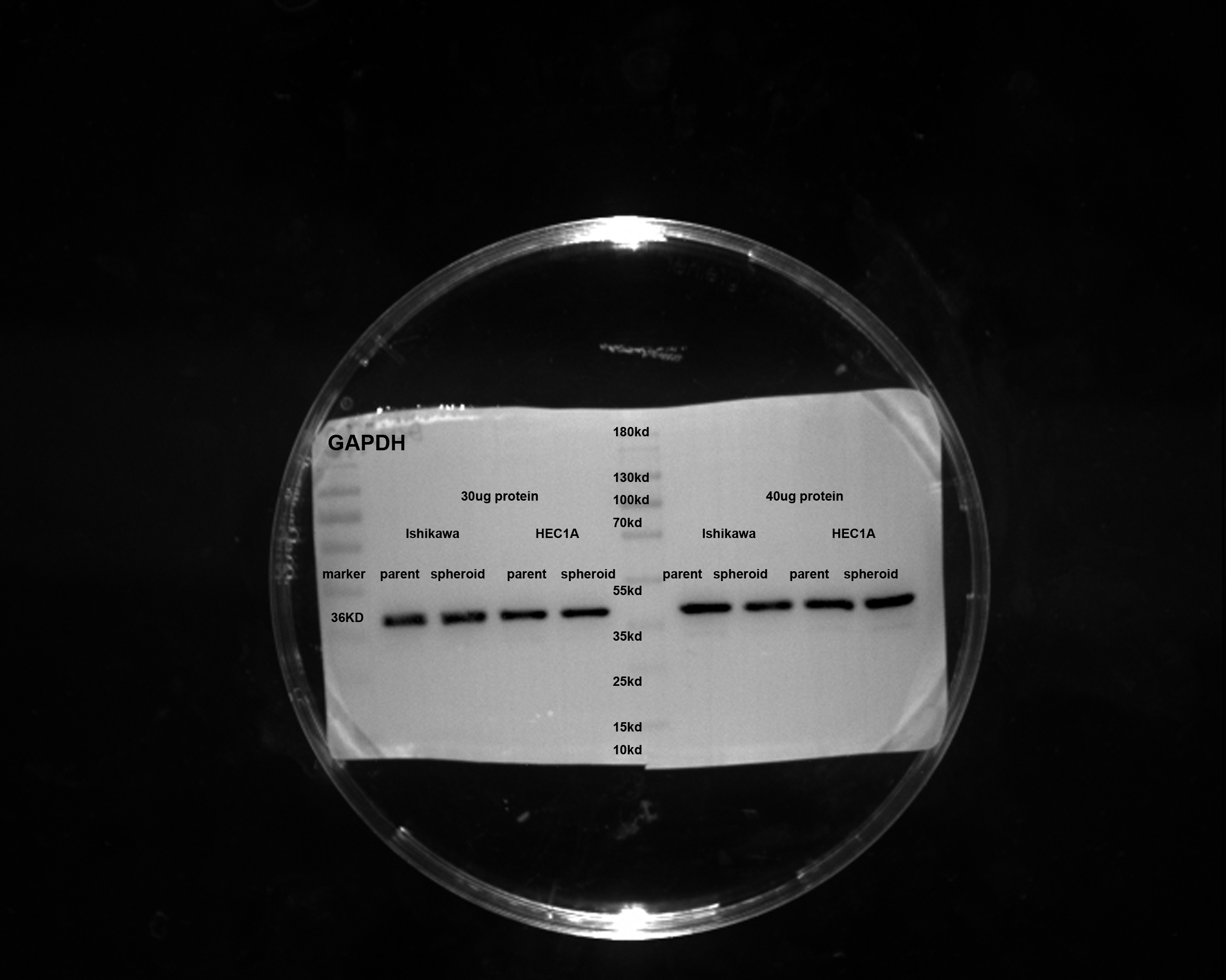


Sfig1-3


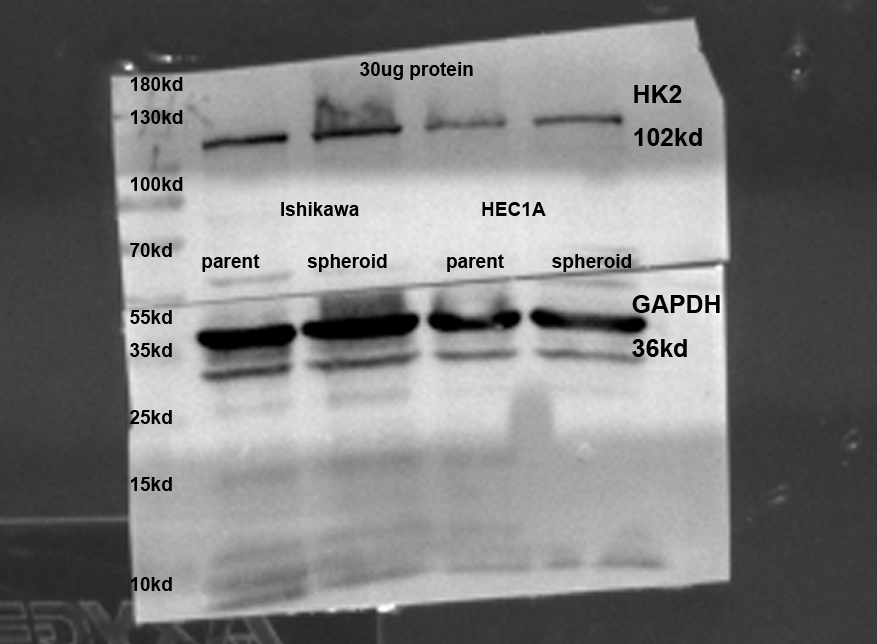


Sfig1-4

1. Original images of Figure 8F


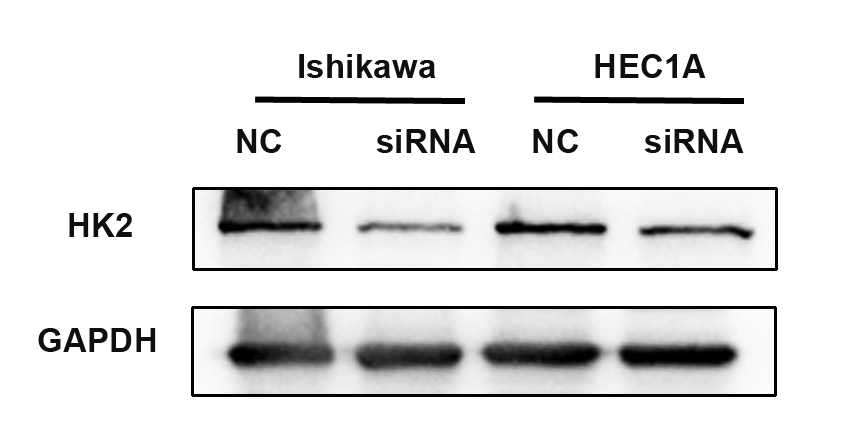


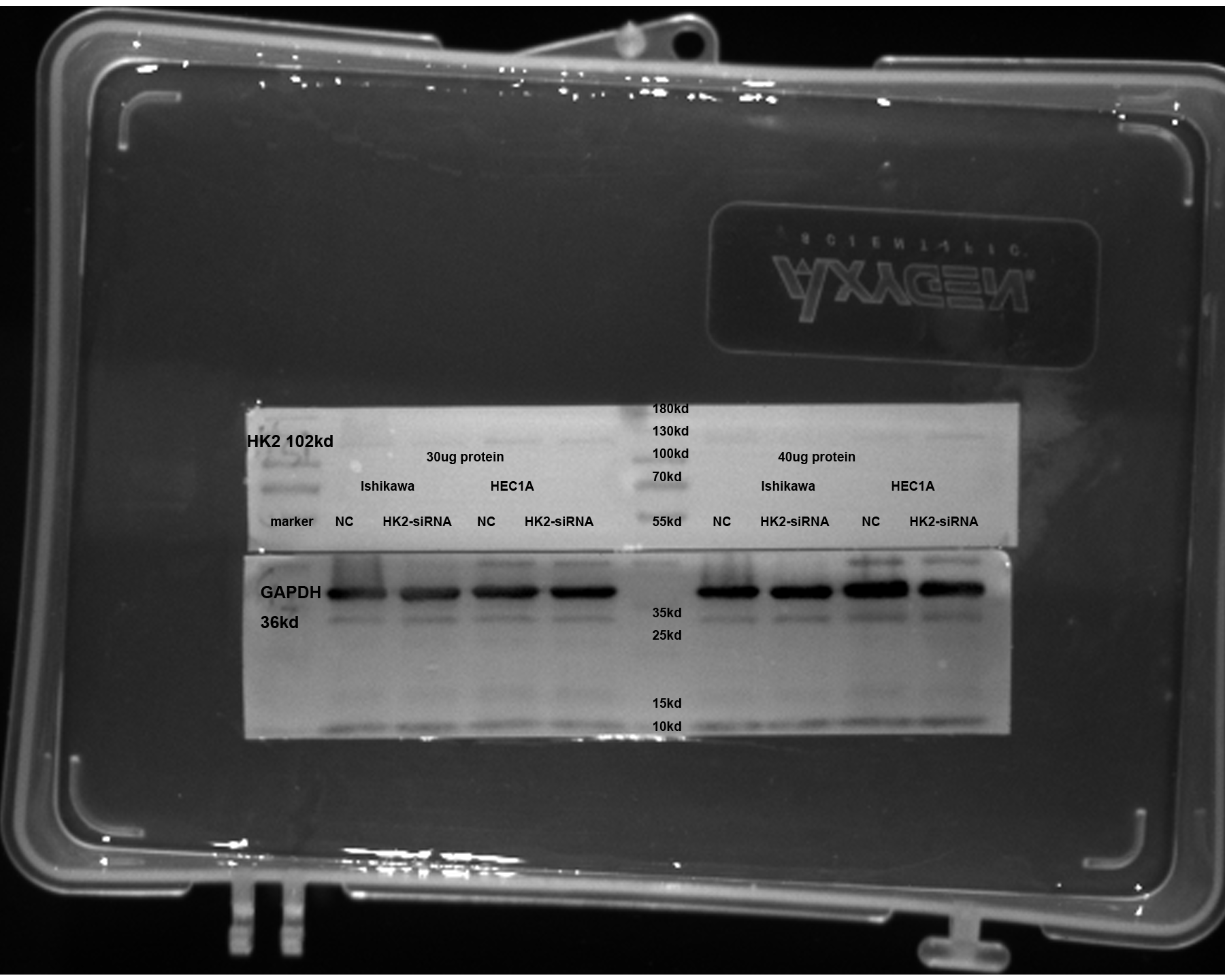


Sfig2-1


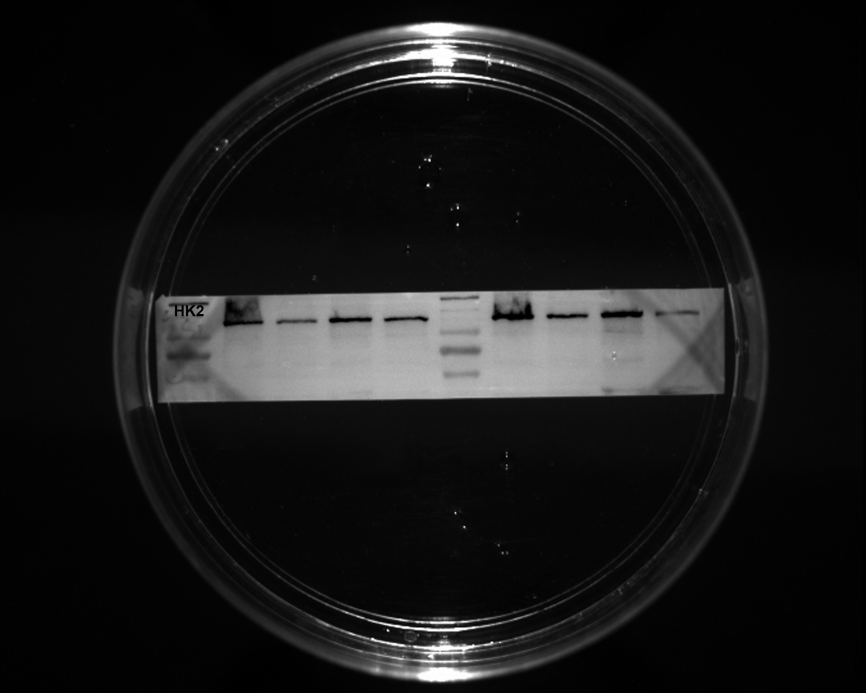


Sfig2-2


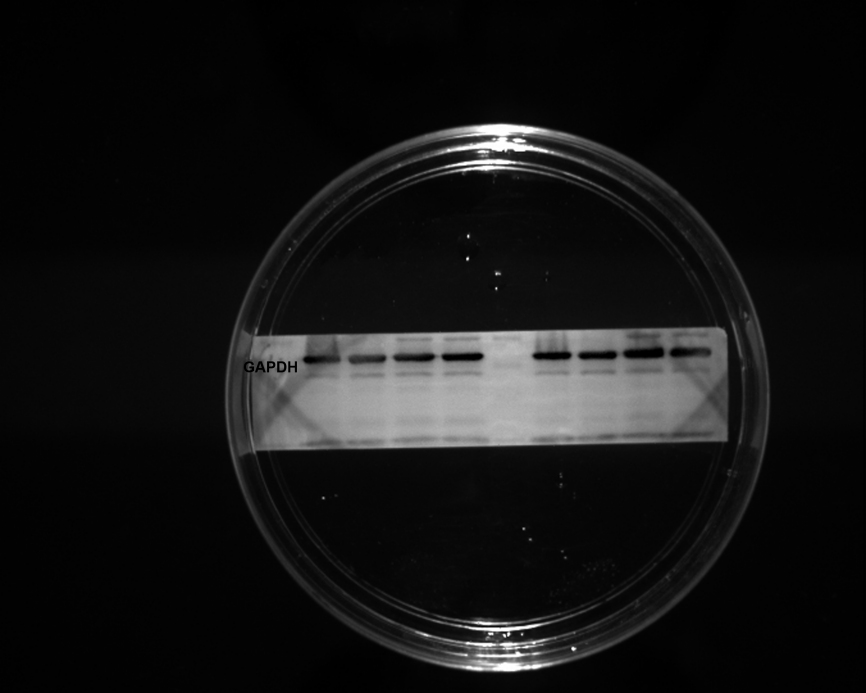


Sfig2-3

Supplement: Supplementary file 1 — Additional file 1. Original uncropped blots of the WB figure in the combined picture. [file 13287_2023_3348_MOESM1_ESM.docx]

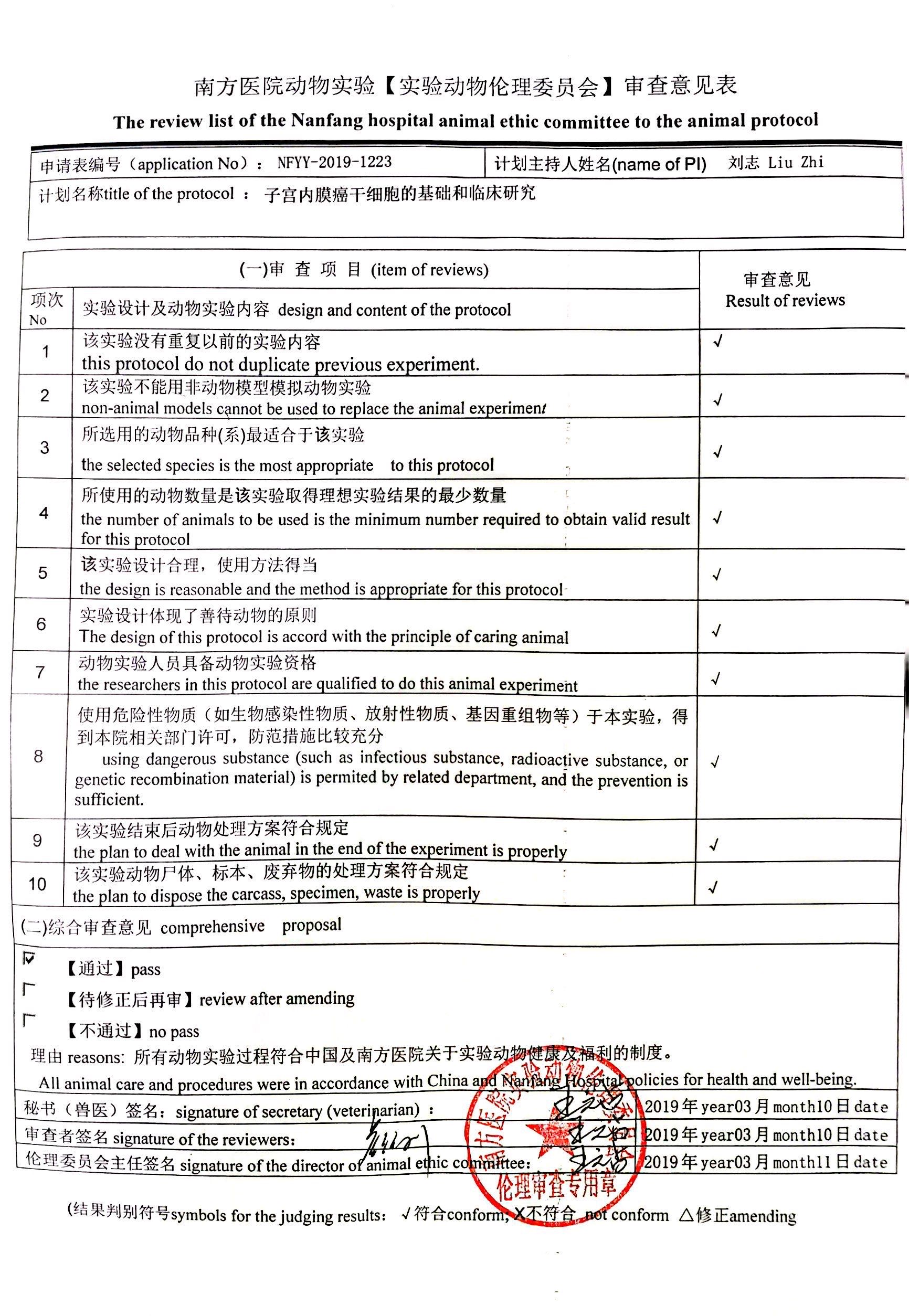

Supplement: Supplementary file 2 — Additional file 2. Ethical approval 1. [file 13287_2023_3348_MOESM2_ESM.jpg]

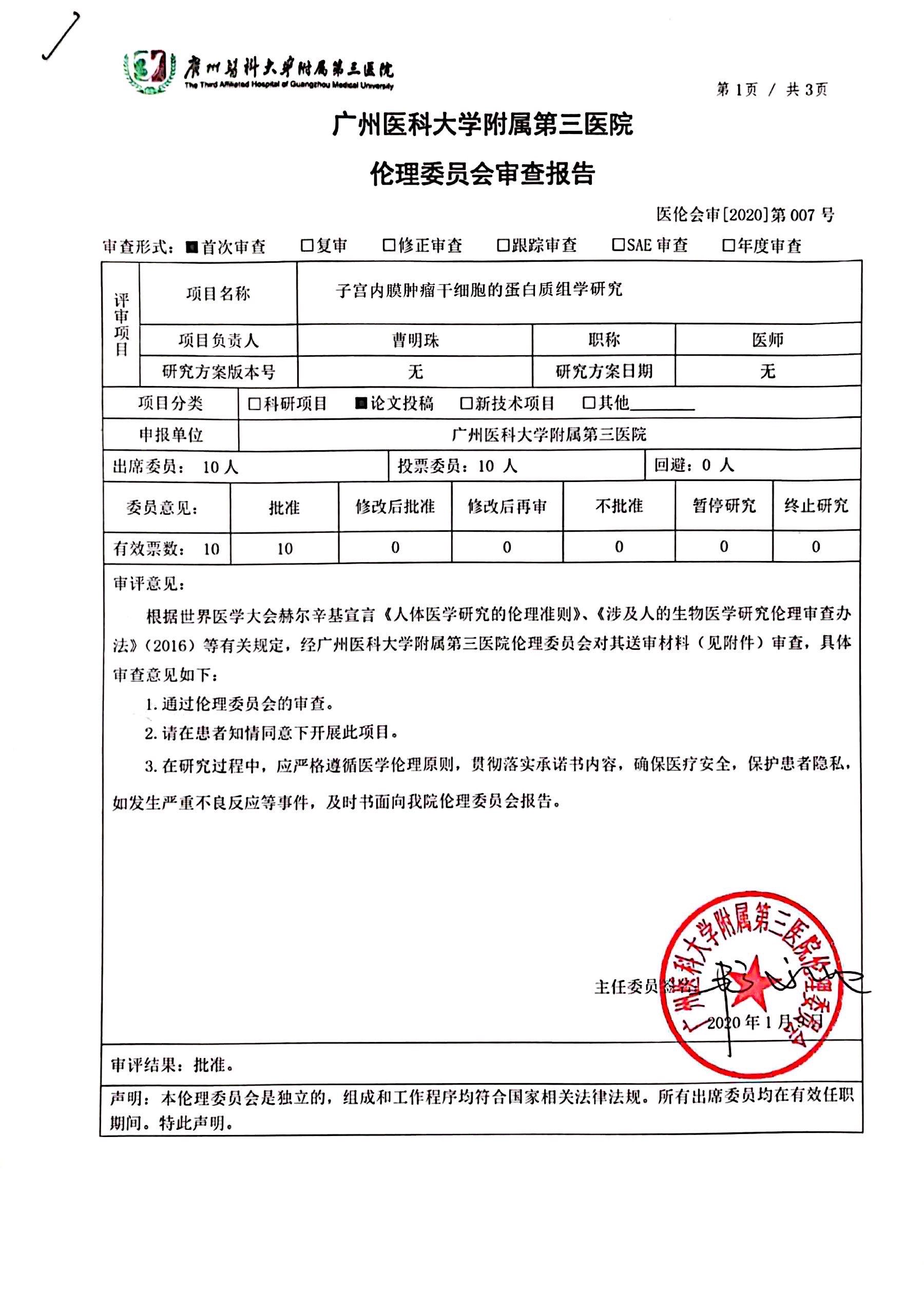

Supplement: Supplementary file 3 — Additional file 3. Ethical approval 2. [file 13287_2023_3348_MOESM3_ESM.jpg]
